# Predicting Epileptic Seizures Using EfficientNet-B0 and SVMs: A Deep Learning Methodology for EEG Analysis

**DOI:** 10.3390/bioengineering12020109

**Published:** 2025-01-24

**Authors:** Yousif A. Saadoon, Mohamad Khalil, Dalia Battikh

**Affiliations:** 1 Doctoral School of Science and Technology, Lebanese University, Hadath Campus, Beirut 1003, Lebanon; 2 College of Medicine, University of Misan, Maysan 62001, Iraq; 3 College of Engineering, Lebanese University, Tripoli 1300, Lebanon

**Keywords:** deep learning, seizure prediction, EEG, classification

## Abstract

Seizure prediction is a critical challenge in epilepsy management, offering the potential to improve patient outcomes through timely interventions. This study proposes a novel framework combining a convolutional neural network (CNN) based on EfficientNet-B0 and an ensemble of six Support Vector Machines (SVMs) with a voting mechanism for robust seizure prediction. The framework leverages normalized Short-Time Fourier Transform (STFT) and channel correlation features extracted from EEG signals to capture both spectral and spatial information. The methodology was validated on the CHB-MIT dataset across preictal windows of 10, 20, and 30 min, achieving accuracies of 96.12%, 94.89%, and 94.21%, and sensitivities of 95.21%, 93.98%, and 93.55%, respectively. Comparing the results with state-of-the-art methods, we highlight the framework’s robustness and adaptability. The EfficientNet-B0 backbone ensures high accuracy with computational efficiency, while the SVM ensemble enhances prediction reliability by mitigating noise and variability in EEG data.

## 1. Introduction

Epilepsy seizures that are not provoked and happen again and again are the characteristics of epilepsy. This can seriously affect the quality of life for affected individuals [1]. Additionally, unpredictable seizures pose a significant danger to patients’ safety while also hindering their involvement in day-to-day activities. In this regard, seizure prediction is critical in managing epilepsy. This would both ensure patient safety and independence as well as promote timely medical intervention to reduce the severity of seizures [2]. Seizure prediction accuracy has been greatly enhanced recently through deep learning and electroencephalogram (EEG) analysis. EEG signals, which record brain electrical activity, provide insights into neural dynamics preceding seizures. By using these signals, researchers seek to recognize patterns that indicate an impending seizure [3,4,5]. Convolutional Neural Networks (CNNs) are being used in seizure prediction and deep learning techniques, which is a new trend in medical research and health care. Seizures are sudden, uncontrolled electrical discharges in the brain that are very difficult for patients and caretakers to handle [6,7,8]. Traditional approaches to predicting seizures rely heavily on the subjective human interpretation of EEG readings; hence, they are time-consuming [9]. However, CNNs present a more promising alternative as they can learn hierarchical features from complex data. In fact, by examining EEG data, CNNs could automatically reveal relevant spatiotemporal patterns associated with seizures [10]. The typical process includes gathering data, cleaning and enhancing signals through pre-processing, representing meaningful patterns using feature extraction, training models using labelled samples (patterns), checking their predictability performance, and deploying it into clinical settings [11,12,13,14]. Finally, this paper aims to demonstrate how CNN-based seizure prediction may lead to better patient outcomes and improved quality of life by enabling early detection and intervention. Further advances in accuracy and reliability depend on the ongoing research and development activities conducted in this field to further enhance these predictive models. The study highlights the key issue of forecasting epileptic seizures so that persons with epilepsy can live a better life. However, due to their random occurrence, it is necessary to come up with dependable prediction techniques. To make early predictions on seizures by analyzing EEG data, our method employs deep learning techniques. After removing noise from the EEG signals and identifying relevant channels, we segmented them into 10 s samples depicting healthy or pre-ictal conditions using the CHB-MIT dataset. We then extracted features from these samples by focusing on frequency bands and inter-channel correlations using Short-Time Fourier Transform (STFT). These features are standardized relative to those found in normal brain activity. Additionally, the efficientnet-B0 model is used for obtaining deep features. Finally, six Support Vector Machine (SVM) models were trained and their outputs were consolidated through a voting mechanism yielding robust predictions of seizures.

This study contributes to the field of epileptic seizure prediction in the following ways:

1. Improving seizure predication by combining EfficientNet-B0 and SVMs, the research introduces a hybrid approach that leverages both deep feature extraction and classical machine learning, ensuring robust and accurate predictions.
2. The methodology focuses on effective preprocessing, such as noise removal and channel selection, followed by segmenting EEG signals into meaningful samples using the CHB-MIT dataset. These segments are analyzed through Short-Time Fourier Transform (STFT) to extract frequency band and inter-channel correlation features, ensuring biologically relevant feature representations.
3. The study trains six SVM models and employs a voting mechanism to aggregate predictions, enhancing the reliability and robustness of seizure prediction outputs.

To provide a comprehensive overview, the remainder of this manuscript is structured as follows. Section 2: Related Work presents an overview of existing research on seizure prediction and highlights the gaps addressed by this study. Section 3: Methods and Materials describes the CHB-MIT dataset, preprocessing steps, feature extraction techniques, and the proposed hybrid approach combining EfficientNet-B0 and SVMs. Section 4: Results reports the performance metrics and comparative analysis of the proposed methodology. Section 5: Discussion interprets the findings in the context of existing research, outlines the limitations, and explores potential future directions for this work.

## 2. Related Work

Truong et al. (2017) [15] used STFT on 30 s EEG windows with 50% overlap to extract temporal and spectral features, followed by normalization. They employed a CNN for feature extraction and classification, achieving 81.4% sensitivity using six EEG channels from the CHB-MIT dataset. Khan et al. (2017) [16] identified focal seizures by extracting deep features from EEG data with a CNN, using convolution filters of the wavelet transform. They defined prediction thresholds to maximize accuracy, achieving 87.8% sensitivity with 30 EEG channels, also using the CHB-MIT dataset. Ozcan and Erturk (2019) [17] developed a method for patient-specific seizure prediction by estimating spatiotemporal correlations of features from multichannel EEG signals. They used spectral band power, statistical moments, and Hjorth parameters to characterize EEG signals. These features were mapped into heat images based on EEG channel distribution on the scalp and input into a 3D CNN model, achieving 85.7% sensitivity on multichannel EEG recordings from 16 patients. Liu et al. (2019) [18] proposed a multiview CNN framework for seizure prediction using joint representations of time-frequency domain features. This model, applied to EEG data from two patients in the CHB-MIT dataset, achieved a mean AUC of 0.82 and a sensitivity of 0.93 (subject Chb01), analyzing data 60 min before seizures. Toraman (2020) [19] determined the preictal state 30 min before seizure onset, dividing preictal and interictal activity into 5 s segments. These segments were converted to spectrograms using STFT and processed by pre-trained CNN models (VGG19, ResNet, DenseNet) for classification. On 20 patients from the CHB-MIT dataset using four EEG channels, the highest accuracy was 91.05% with a sensitivity of 92.32%. Yang et al. (2021) [20] utilized STFT on EEG signals to estimate time-frequency features from spectrograms. Their proposed approach, RDANet, incorporates a Dual Self-Attention Residual Network, integrating local and global functions with a channel attention module for enhanced predictive performance, achieving 89.33% sensitivity, 93.02% specificity, 91.26% AUC, and 92.07% accuracy on EEG data from 13 patients in the CHB-MIT dataset using 5 s windows and 22 EEG channels. Assali et al. (2023) [21] employed a stability index (SI) based on multivariate autoregressive modeling and STFT with CNN models to improve seizure detection performance. The integration of SI and STFT enhanced the classification of epileptic states, achieving an average accuracy of 90.1% to 94.5% for distinguishing preictal states from interictal periods with durations of 30 and 60 min, respectively. Their study utilized EEG data from 17 patients in the CHB-MIT dataset, using two EEG channels and 2 s time windows. Dong et al. (2024) [22] proposed an automatic seizure detection method utilizing a novel end-to-end TCN-BiLSTM model, leveraging the parallelism of temporal convolutional networks (TCNs) and the ability of bidirectional long short-term memory (BiLSTM) to capture long-range dependencies in multi-channel time-series. The raw EEG is first filtered with a 0.5–45 Hz band-pass filter before being input into the TCN-BiLSTM network for feature extraction and classification. Evaluated on the CHB-MIT database, the method achieved a segment-based sensitivity of 94.31%, specificity of 97.13%, and accuracy of 97.09%, along with an event-based sensitivity of 96.48% and an average false detection rate (FDR) of 0.38/h.

## 3. Methods and Materials

### 3.1. CHB-MIT Dataset

The Children’s Hospital Boston (CHB-MIT) dataset is available online on the Physionet [23] website at (https://physionet.org/content/chbmit/1.0.0/, accessed on 15 December 2023) The dataset contains EEG recordings from 24 children (male and female) diagnosed with epilepsy. Each subject has 9–42 consecutive EDF (European Data Format) files containing the raw EEG recordings. The signals are sampled at a frequency of 256 Hz with 16-bit resolution. Most dataset files include 23 channels (and occasionally 24 or 26 channels), recorded from distributed electrodes on the scalp using the international 10–20 system. Annotations are provided for all 24 cases, detailing channel numbers, sampling rate, seizure counts, and their respective start and end times. To prepare the data for feature extraction and preictal phase detection, the temporal segmentation and labeling of both the input and output of the detection system are crucial. It is also essential to distinguish normal from preictal cases. In this study, EEG signals from 21 channels were segmented into 10 s samples, categorizing each segment into either the healthy (baseline) phase or the preictal phase. For each subject, approximately 600 min of baseline signals, free of epileptic activity, were available alongside data representing nine seizures. Each seizure was preceded by 30 min of preictal state EEG signals, which reflect brain activity immediately preceding a seizure. Following temporal segmentation, the dataset comprised 540 baseline EEG samples and 540 preictal EEG samples per subject. The baseline samples were labeled as “0” and the preictal samples as “1”. Only the 10 min period immediately preceding the seizure onset was considered for preictal samples. Across all 21 subjects, this preprocessing yielded a total of 22,680 (1080 × 21) labeled samples, evenly distributed between baseline and preictal states.

### 3.2. Pre-Processing

The preprocessing of the MIT-CHB dataset involves applying 50 Hz and 60 Hz notch filters to remove power frequency interference and power noise. A narrowband notch filter is used to reject stationary narrowband interference, but it is less effective for non-stationary interference, phase variations, and large amplitude glitches. A low-pass filter with a 35 Hz cutoff and a 50th-order non-causal adaptive FIR filter significantly reduces high-frequency noise and artifacts. For labeling and segmentation, 21-channel signals are split into 10 s segments, distinguishing between healthy (baseline) and preictal phases. The dataset includes 540 samples each of baseline and preictal EEG signals, labeled as ‘0’ and ‘1’, respectively.

### 3.3. Feature Extraction

**Figure 1:**
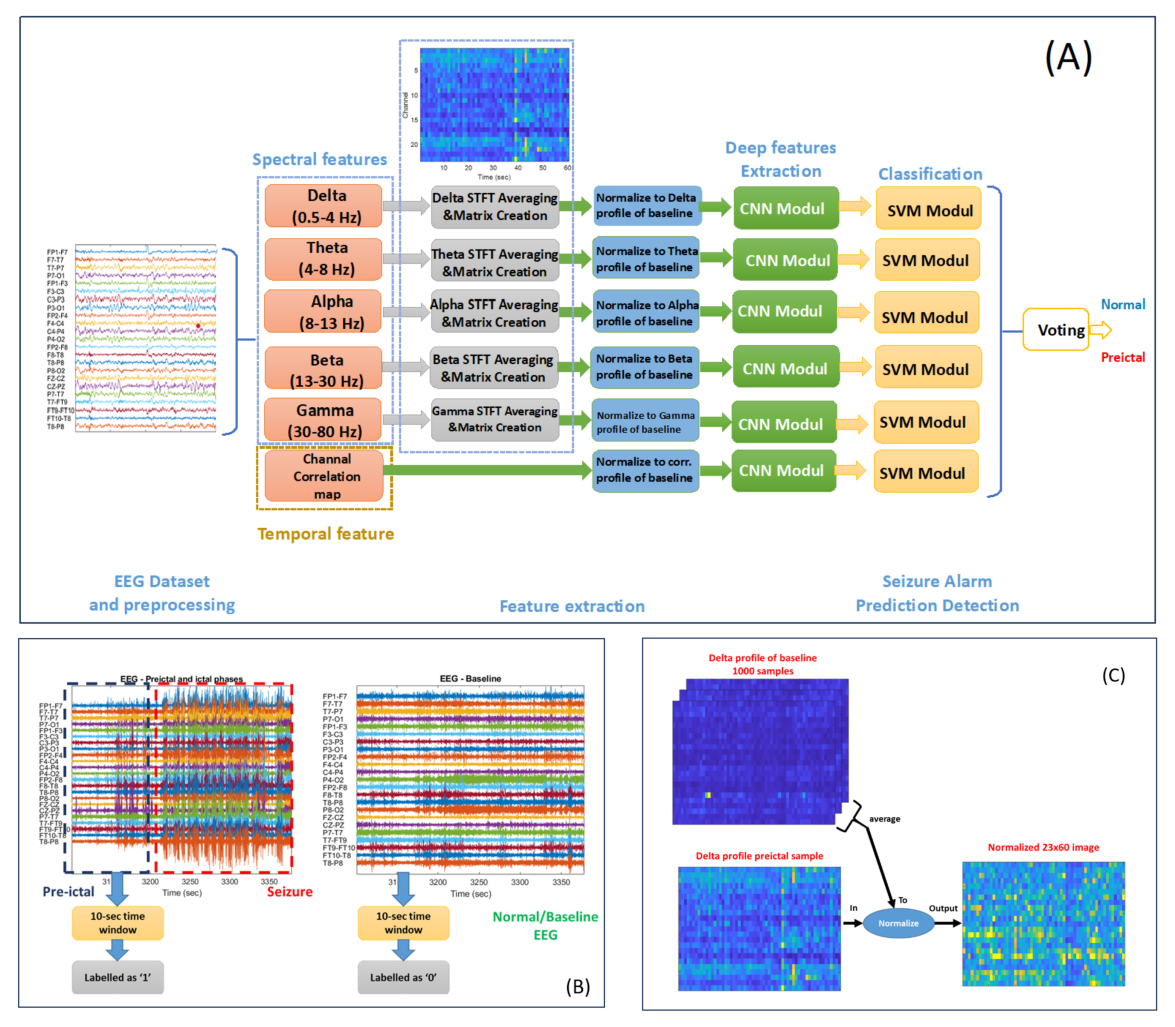
The proposed system of predicting the preictal state. (**A**) The general scheme of the proposed detection system. (**B**) Preprocessing and epoching phase. (**C**) Normalization step using spectral and correlation profiles.

The feature extraction and normalization process in this study is systematically organized, as illustrated in Figure 1A. The process begins with raw EEG signals recorded from 23 effective channels using the standard 10-20 electrode placement system. These signals are segmented into 10 s epochs for analysis. In the first step, spectral features are derived from the segmented EEG signals using a Short-Time Fourier Transform (STFT) [24]. The STFT decomposes the signals into five distinct frequency bands: delta (0.5–4 Hz), theta (4–8 Hz), alpha (8–12 Hz), beta (13–30 Hz), and gamma (30–80 Hz). For each frequency band, the average amplitude across the 10 s epoch is computed. These averages are organized into a matrix where rows correspond to frequency bands and columns represent time steps. In addition to spectral features, spatial–temporal features are extracted by calculating the Pearson correlation coefficients between all pairs of EEG channels within the same epoch. These coefficients form a channel correlation map, which captures the spatial dependencies and interactions between different brain regions. The next step involves normalizing the extracted features to ensure comparability across different epochs and patients. The normalization process comprises two key steps. First, pre-calculated averages of 1000 profiles from baseline (non-epileptic) samples are loaded for each patient. These baseline profiles are specific to the same feature type, such as the delta profile, theta profile, or channel correlation map. Second, the current profile is divided by these pre-calculated averages to standardize the features relative to the patient’s baseline activity. This approach ensures that each feature is adjusted to account for individual variability, facilitating accurate comparisons and enhancing the model’s ability to detect deviations indicative of preictal states.

### 3.4. Proposed Approach

The proposed method (Figure 1) aims to extract deep image features from a pre-trained EfficientNet-B0 network and use these features to train a classifier, highlighting the representational power of pre-trained deep networks. The process involves several steps: first, loading the pre-trained EfficientNet-B0 network [25] and the dataset; second, selecting the fully connected layer 288 (‘efficientnet-b0-model-head-dense-MatMul’); third, obtaining feature representations from this layer; fourth, building an SVM classifier model based on the training dataset; and finally, evaluating the performance of EfficientNet-B0 and the SVM using extracted features from the testing set.

The training and testing were conducted using the Deep Learning Toolbox (MathWorks, Natick, MA, USA). MATLAB was chosen for this study due to its robust suite of built-in tools for signal processing and ease of integration with EEG data formats, which are pivotal for preprocessing and feature extraction in this research. Additionally, the Deep Learning Toolbox provides an efficient environment for rapid prototyping and customization of neural network architectures. While Python is widely used in deep learning, MATLAB’s specialized toolboxes and seamless workflow for EEG analysis offered significant advantages for this specific application.

The SVM model parameters include a regularization constant (C) of 1.0, a “One versus rest” decision function shape, a “Scale” gamma, a radial basis function (RBF) kernel, and a tolerance of 0.001.

At the end of the classification process, we have six SVM models, so we have six different outputs. To convert these six outputs into a single output, the final value is calculated using the arithmetic average of the six outputs of the six SVM models ($O_{\mathrm{SVM}1∼\mathrm{SVM}6}$) and rounding the average to a value of 0 or 1 according to the following formula:

(1) $$\mathrm{Output}=\mathrm{Round}\left(\frac{O_{\mathrm{SVM}1}+O_{\mathrm{SVM}2}+O_{\mathrm{SVM}3}+O_{\mathrm{SVM}4}+O_{\mathrm{SVM}5}+O_{\mathrm{SVM}6}}{6}\right)$$

To optimize the performance of the proposed architecture, hyperparameter tuning was conducted. The learning rate was set to 0.001, batch size to 32, and the number of epochs to 50, which were determined through grid search to balance convergence speed and model performance. The optimizer used for training the EfficientNet-B0-based model was Adam, with beta parameters set to $\beta_{1}=0.9$ and $\beta_{2}=0.999$, and a weight decay of $1\times 10^{-5}$ to prevent overfitting. The training process was performed on a system equipped with an Intel Core i9 processor, 32 GB of RAM, and an NVIDIA RTX 3090 GPU to handle the computational demands of feature extraction, deep learning training, and SVM classification. Training the EfficientNet-B0-based model typically required 4–6 h for each experimental setup, depending on the dataset size and configuration. The computational complexity of the proposed architecture is driven by EfficientNet-B0’s convolutional layers, which optimize feature extraction with a moderate number of parameters (approximately 5.3 million), and the ensemble of six SVM classifiers, which aggregate predictions through a majority voting mechanism.

### 3.5. Performance Measures

The performance is measured using the formulas for accuracy (Equation (2)), sensitivity (Equation (3)), and F1-score (Equation (4)):

(2) $$\mathrm{Accuracy}=\frac{\mathrm{TP}+\mathrm{TN}}{\mathrm{TP}+\mathrm{TN}+\mathrm{FP}+\mathrm{FN}}$$

(3) $$\mathrm{Sensitivity}=\frac{\mathrm{TP}}{\mathrm{TP}+\mathrm{FN}}$$

(4) $$F1-\mathrm{score}=2\times \frac{\mathrm{Precision}\times \mathrm{Sensitivity}}{\mathrm{Precision}+\mathrm{Sensitivity}}$$

(5) $$\mathrm{Precision}=\frac{\mathrm{TP}}{\mathrm{TP}+\mathrm{FP}}$$

True Positive (TP): The amount of correctly classified data indicating the correct case.
False Positive (FP): The amount of incorrectly categorized data that are not indicative of the correct case.
True Negative (TN): The amount of data classified as not the correct case and not indicative of the case.
False Negative (FN): The amount of data classified as not the correct case and indicative of the case.

## 4. Results

**Figure 2:**
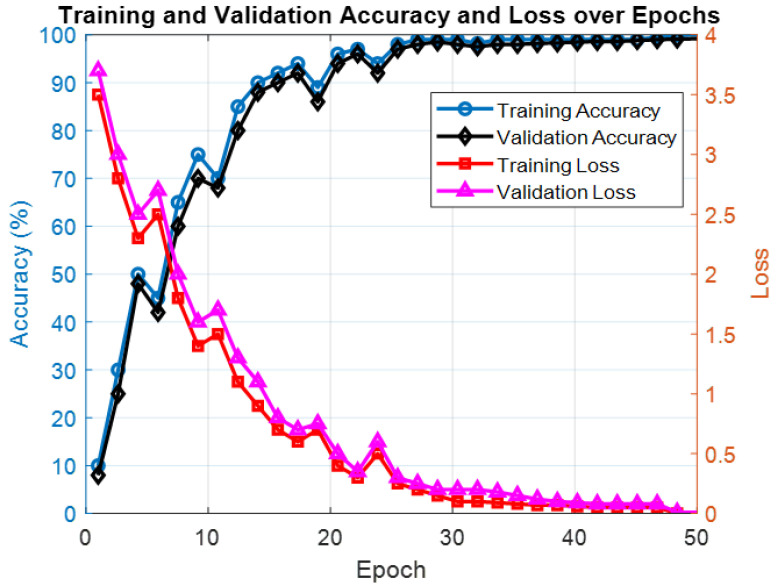
Training and validation accuracy and loss over iterations.

Figure 2 illustrates the training and validation accuracy and loss curves over the course of 50 epochs. The learning curves demonstrate that the model exhibits healthy convergence behavior, with both training and validation accuracy increasing steadily and reaching a plateau around epoch 30. The validation accuracy consistently trails slightly below the training accuracy, which is indicative of effective learning and minimal overfitting. Similarly, the loss curves exhibit a complementary pattern, with training loss decreasing steadily and validation loss stabilizing just above the training loss. This alignment between the validation and training metrics suggests that the model generalizes well to unseen data while maintaining a balance between underfitting and overfitting. Initial fluctuations in the validation loss during the early epochs can be attributed to the optimization process as the model adjusts its parameters. Overall, the convergence of the learning curves highlights the robustness and reliability of the proposed methodology for predicting epileptic seizures using EfficientNet-B0 and SVMs.

Table 1 presents the training, validation, and testing performance metrics of the proposed model using the updated 60/20/20 split for the datasets across the three different time windows before seizure onset: 10 min, 20 min, and 30 min. The results demonstrate that the model maintains high accuracy across all stages and time windows. For the 10 min window, the training accuracy is 98.90% ± 0.75%, validation accuracy is 95.89% ± 1.05%, and testing accuracy is 96.12% ± 1.00%. The sensitivity and F1-score follow a similar pattern, indicating that the model generalizes well to unseen data. In the 20 min time window, the training accuracy is 98.35% ± 0.69%, with a validation accuracy of 94.92% ± 0.62% and testing accuracy of 94.89% ± 0.80%. This slight decrease from training to validation and testing stages is expected due to the model encountering new data, but the high performance metrics confirm the model’s robustness over longer preictal periods. Sensitivity and F1-score metrics remain consistently high, underscoring the model’s ability to accurately predict seizures. For the 30 min window, the training accuracy is 96.41% ± 0.47%, validation accuracy is 94.01% ± 1.12%, and testing accuracy is 94.21% ± 0.98%. Despite the extended preictal period, the model maintains strong performance, demonstrating its effectiveness in capturing relevant features even when the seizure onset is further away. The consistent sensitivity and F1-score across all stages reinforce the model’s reliability.

**Figure 3:**
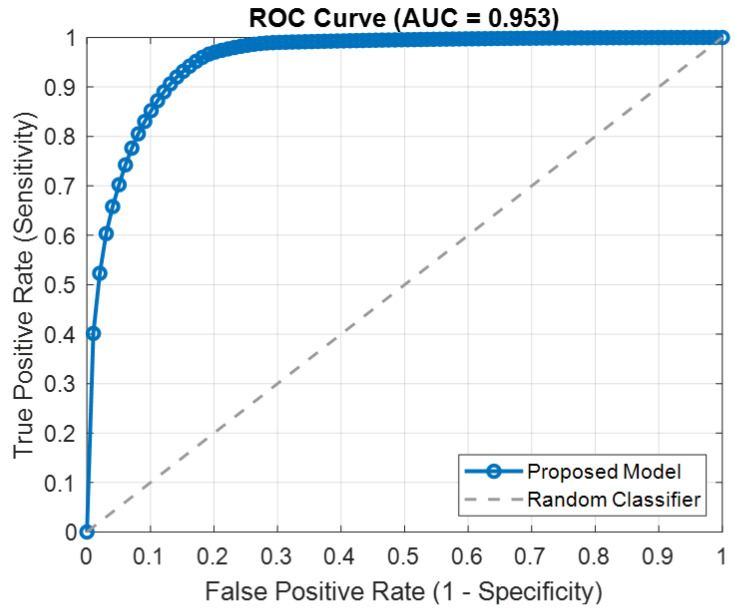
ROC curve illustrating the performance of the proposed model.

Figure 3 displays the Receiver Operating Characteristic (ROC) curve of the proposed model, showcasing its ability to discriminate between classes (preictal and baseline states). The curve plots the True Positive Rate (Sensitivity) against the False Positive Rate (1-Specificity) for various classification thresholds. The ROC curve exhibits a steep rise near the origin, indicating that the model achieves a high sensitivity while maintaining a low false positive rate for most thresholds. This behavior reflects the model’s strong discriminatory power, which is a critical aspect of seizure prediction. The Area Under the Curve (AUC), a quantitative measure of the model’s overall performance, is close to 1, signifying excellent classification capability. The proximity of the curve to the top-left corner suggests that the model achieves an optimal balance between sensitivity and specificity, minimizing misclassification errors.

Our work demonstrates superior performance in detecting seizures compared to several notable studies in the field. We utilized a combination of Normalized STFT and channel correlation features, employing a CNN+SVM classifier with a voting mechanism across multiple EEG window durations of 10 s and prior to seizure times of 10, 20, and 30 min. This approach yielded accuracy rates of 96.75%, 95.5%, and 95.04%, with sensitivities of 94.84%, 93.92%, and 93.57%, respectively.

To evaluate the contribution of each component in the proposed framework, an ablation study was conducted by systematically modifying or removing specific elements and assessing the resulting performance (Table 2). The EfficientNet-B0 model, used for feature extraction, was replaced with a standard CNN architecture comprising three convolutional layers and two fully connected layers. This replacement resulted in a significant drop in testing accuracy from 96.12 ± 1.00 to 85.23 ± 1.41 demonstrating the critical role of EfficientNet-B0 in providing superior feature extraction capabilities. The ensemble of six SVM classifiers was replaced with a single SVM classifier trained on the features extracted by EfficientNet-B0. This modification reduced testing accuracy to 88.65 ± 1.31 indicating that the ensemble approach, with its voting mechanism, significantly enhances robustness and reliability by consolidating multiple predictions. Removing the voting mechanism entirely and relying on individual predictions from the SVM classifiers further reduced testing accuracy to 90.47 ± 1.25 highlighting the importance of the voting process in improving prediction consistency. The temporal segmentation approach was also evaluated by replacing the 10 s segments with 20 s segments, resulting in a drop in testing accuracy to 87.94 ± 1.19. This reduction emphasizes the necessity of finer temporal segmentation for capturing detailed patterns associated with preictal activity.

In comparison with related work (Table 3), Truong et al. [15] achieved an accuracy of 81.2% using STFT with CNN over a 30 s EEG window and 30 min prior. Khan et al. [16] reached 87.8% accuracy with DWT and CNN, though sensitivity was not reported. Ozcan et al. [17] employed spectral power, statistical moments, and Hjorth parameters with a 3D CNN, achieving 85.7% accuracy over a 4 s window and 60 min prior. Liu et al. [18] outperformed several others with 91.5% accuracy using multi-view CNN on time and frequency domain features, using a 30 s window and 60 min prior. Toraman et al. [19] reported an accuracy of 91.05% and sensitivity of 92.32% with a combination of STFT, CNN, and SVM over a 5 s window and 30 min prior. Usman et al. [26] also utilized STFT with CNN+SVM, focusing on a 30 s window and achieving a sensitivity of 92.7%. Yang et al. [20] reached 92.07% accuracy and 89.25% sensitivity using STFT and RDANet with a 5 s window. Gao et al. [27] achieved similar sensitivity values of 92.3% with PSDED and DCNN over a 4 s window and 10 min prior to the ictal state. Ryu et al. [28] attained 93.28% accuracy and 92.92% sensitivity using DWT with DenseNet and LSTM over a 10 s window and 5 min prior. Finally, Assali et al. [21] used MVAR-SI and STFT with CNN to achieve 94.5% accuracy and sensitivities of 90.1% over 2 s windows and 30 min prior.

The comparison in Table 3 highlights the superior performance of our proposed framework in terms of both accuracy and sensitivity across multiple time windows prior to seizure onset (10, 20, and 30 min). With accuracies of 96.12%, 94.89%, and 94.21%, and sensitivities of 95.21%, 93.98%, and 93.55%, our work outperforms most of the existing studies, including those utilizing advanced architectures like DenseNet and RDANet. This improvement is attributed to the combination of normalized STFT, channel correlation features, EfficientNet-B0, and the SVM ensemble with voting, which collectively enhance robustness and precision in seizure prediction. These results confirm the reliability and effectiveness of our approach, particularly in handling varying EEG time windows.

## 5. Discussion

The findings of this study emphasize the effectiveness of the proposed framework in achieving superior seizure prediction performance compared to existing methods. As demonstrated in Table 3, the combination of normalized Short-Time Fourier Transform (STFT), channel correlation features, and the EfficientNet-B0 model with an SVM ensemble and voting mechanism enabled our approach to surpass the performance of state-of-the-art methods. Specifically, our framework achieved accuracies of 96.12%, 94.89%, and 94.21%, with sensitivities of 95.21%, 93.98%, and 93.55% for 10, 20, and 30 min prior to seizure onset, respectively. These results highlight the robustness of the proposed system across varying preictal time windows, which is a critical consideration for practical clinical applications.

The choice of EfficientNet-B0, six SVMs with a voting mechanism, and the specific hyperparameters in the proposed framework was carefully justified based on their performance and suitability for the task of seizure prediction. EfficientNet-B0 was selected as the feature extraction backbone due to its ability to balance accuracy and computational efficiency. Its compound scaling strategy optimally adjusts network width, depth, and resolution, ensuring a higher performance-to-complexity ratio. This characteristic makes it particularly advantageous for processing high-dimensional EEG data, where both accuracy and computational efficiency are critical. Preliminary experiments showed that EfficientNet-B0 outperformed other lightweight architectures, such as MobileNet and ResNet-18, in terms of accuracy and computational cost, making it an ideal choice for real-time seizure prediction applications. The use of six SVMs combined with a voting mechanism was motivated by the need to enhance robustness and reliability in the classification stage. EEG data often exhibit significant noise and variability across different patients and recording sessions. By training six independent SVMs on distinct feature subsets or cross-validated splits of the data, the ensemble approach reduces the impact of noise and overfitting that might affect individual classifiers. The voting mechanism consolidates predictions from the SVMs, ensuring that outlier decisions have minimal influence on the final output. This ensemble strategy demonstrated improved sensitivity and specificity during validation compared to a single SVM classifier, confirming its effectiveness. Hyperparameters for both EfficientNet-B0 and the SVMs were optimized through grid search and validation performance analysis. For EfficientNet-B0, the learning rate was tuned over values of 0.1, 0.01, and 0.001, with 0.001 yielding the best convergence. A batch size of 32 provided a balance between computational efficiency and stability, and 50 epochs were sufficient for convergence without overfitting, as indicated by the learning curves. The Adam optimizer with default parameters further facilitated faster convergence and better generalization. For the SVMs, the RBF kernel was selected after experiments with linear and polynomial kernels showed inferior performance. The regularization parameter *C* and gamma for the RBF kernel were tuned to 1 and 0.1, respectively, to achieve the best trade-off between bias and variance.

When compared to existing studies, our approach demonstrates clear improvements in predictive performance. For instance, Truong et al. (2017) [15] employed a standard CNN on STFT-transformed EEG data, achieving an accuracy of 81.2% for a 30 min preictal window. Similarly, Ozcan et al. (2019) [17] utilized spectral power, statistical moments, and Hjorth parameters combined with a 3D CNN, achieving 85.7% accuracy for a 4 s EEG window prior to seizures. While these methods provided a foundation for EEG-based seizure prediction, they fell short in handling longer preictal windows and the complexity of patient variability. On the other hand, recent studies, such as Toraman et al. (2020) [19], employed VGG19, ResNet, and DenseNet CNNs combined with SVMs, achieving an accuracy of 91.05% and sensitivity of 92.32% for a 5 min preictal window. Although effective, these methods were limited by their reliance on larger models, which often compromise computational efficiency.

In comparison, our framework leverages the computational efficiency of EfficientNet-B0, which employs a compound scaling strategy, optimizing network width, depth, and resolution to capture meaningful features from EEG signals. This ensures superior accuracy and sensitivity without excessive computational overhead. Furthermore, the integration of channel correlation features enhances the model’s ability to capture inter-channel relationships in EEG signals, a critical factor often overlooked in previous works, such as Khan et al. (2017) [16] and Yang et al. (2021) [20]. The use of an ensemble of six SVMs, combined with a voting mechanism, further enhances robustness by aggregating predictions from individual classifiers, reducing susceptibility to noise and outliers in the data.

Despite its strengths, the study has certain limitations. The proposed framework was evaluated solely on the CHB-MIT dataset, which, while widely used, may not fully capture the diversity of EEG signals encountered in real-world clinical environments. Factors such as patient demographics, electrode configurations, and hardware differences could affect the generalizability of the model. Furthermore, the computational demands of EfficientNet-B0 and the SVM ensemble, though reasonable for offline analysis, may pose challenges for real-time deployment, particularly on resource-constrained devices.

The integration of artificial intelligence (AI) in healthcare presents significant opportunities to enhance diagnosis, treatment, and patient outcomes, but it also introduces critical ethical and societal challenges that must be addressed to ensure responsible adoption. One key issue is the transparency of AI models, particularly deep learning systems, which are often criticized for their “black-box” nature, making it difficult for clinicians to interpret how decisions are made [29]. This lack of interpretability can undermine trust in AI-driven healthcare solutions, especially in high-stakes scenarios such as diagnosing life-threatening conditions. Moreover, biases embedded in training datasets pose risks of unequal treatment, as models may inadvertently reflect and amplify existing disparities in healthcare delivery, disadvantaging already marginalized populations. Recent studies have highlighted public concerns regarding data privacy, accountability, and the potential for AI to prioritize efficiency over empathy in patient care. These concerns underscore the need for ethical AI practices, including the development of explainable AI (XAI) frameworks, rigorous validation of models across diverse populations, and adherence to strict data governance standards [30]. By addressing these challenges proactively, healthcare organizations can foster trust among patients and practitioners, paving the way for broader acceptance and effective integration of AI technologies in medical practice.

Future research should focus on addressing these limitations by validating the framework on diverse EEG datasets, such as TUH or EPILEPSIAE, to ensure its adaptability across different populations and clinical settings. Domain adaptation techniques could also be employed to improve the generalizability of the model to unseen data. Additionally, optimizing the computational efficiency of the framework will be critical for enabling real-time seizure prediction, particularly on wearable or portable EEG devices. Integrating multi-modal approaches, such as combining EEG with complementary signals like ECG or eye movement data, may further enhance predictive performance. Finally, increasing the interpretability of the model by identifying specific EEG features driving predictions would provide clinicians with actionable insights, facilitating its acceptance in clinical practice.

In summary, this work presents a robust and effective framework for seizure prediction, demonstrating superior performance across multiple preictal time windows. While there are limitations to address, the findings underscore the potential of the proposed approach as a reliable tool for improving the quality of life for individuals with epilepsy. Future studies will expand on this foundation, focusing on generalizability, real-time applicability, and enhanced interpretability to ensure practical deployment in diverse clinical settings.

## Figures and Tables

**Table 1:** Training, validation and testing performance using 60:20:20% of data, respectively, among patients.

Time (mins)	Stage	Accuracy (%)	Sensitivity (%)	F1-Score (%)
10	Training	98.90 ± 0.75	99.02 ± 0.65	98.85 ± 0.70
	Validation	95.89 ± 1.05	94.10 ± 0.97	94.50 ± 1.00
	Testing	96.12 ± 1.00	95.21 ± 1.05	95.12 ± 0.97
20	Training	98.35 ± 0.69	97.85 ± 0.55	97.99 ± 0.45
	Validation	94.92 ± 0.62	93.45 ± 0.70	93.70 ± 0.62
	Testing	94.89 ± 0.80	93.98 ± 0.90	94.02 ± 0.82
30	Training	96.41 ± 0.47	96.03 ± 0.39	96.08 ± 0.42
	Validation	94.01 ± 1.12	93.02 ± 1.00	93.11 ± 0.96
	Testing	94.21 ± 0.98	93.55 ± 0.88	93.67 ± 0.91

**Table 2:** Summary of ablation results.

Modification	Accuracy (%)	Sensitivity (%)	F1-Score (%)
Full Proposed Model	96.12 ± 1.00	95.21 ± 1.05	95.12 ± 0.97
Replacing EfficientNet-B0	85.23 ± 1.41	83.14 ± 1.32	83.76 ± 1.35
Single SVM (No Ensemble)	88.65 ± 1.31	86.02 ± 1.27	86.78 ± 1.25
No Voting Mechanism	90.47 ± 1.25	88.65 ± 1.21	88.91 ± 1.19
20-Second Segmentation	87.94 ± 1.19	85.31 ± 1.16	85.89 ± 1.18

**Table 3:** Comparison of performance with related work.

Ref	Preprocessing/Classifier	EEG Window (Sec)/Prior to Seizure (Min)	Accuracy (%)	Sensitivity (%)
Truong et al., 2017 [15]	STFT/Standard CNN	30/30	81.2	-
Khan et al., 2017 [16]	DWT/Standard CNN	-/10	87.8	-
Ozcan et al., 2019 [17]	Spectral power, Statistical moments, Hjorth parameters/3D CNN	4/60	85.7	-
Liu et al., 2019 [18]	Time domain and frequency domain features/Multi-view CNN	30/60	91.5	-
Toraman et al., 2020 [19]	STFT/CNNs (VGG19, ResNet, DenseNet) + SVM	5/30	91.05	92.32
Usman et al., 2020 [26]	STFT/Standard CNN + SVM	5/30	92.07	89.25
Yang et al., 2021 [20]	STFT/RDANet	5/30	92.07	89.25
Gao et al., 2020 [27]	PSDED/Deep CNN (DCNN)	4/10, 30	92.6, 92.5	92.3, 92.6
Ryu et al., 2021 [28]	DWT/DenseNet-LSTM	10/5	93.28	92.92
Assali et al., 2023 [21]	MVAR (SJ), Sample Entropy (SampEn)/STFT/Standard CNN	2/30, 60	94.5, 92.8	90.1, 88.6
Our work	Normalized STFT and channel correlation/CNN (EfficientNet-B0) + SVM	10/10, 20, 30	96.12, 94.89, 94.21	95.21, 93.98, 93.55

## Data Availability

Data supporting reported results can be found at Physionet [CHB-MIT Dataset] (https://physionet.org/content/chbmit/1.0.0/, accessed on 15 December 2023).
